# Pitfalls in using ML to predict cognitive function performance

**DOI:** 10.21203/rs.3.rs-4745684/v1

**Published:** 2024-08-17

**Authors:** Gianna Kuhles, Sami Hamdan, Stefan Heim, Simon Eickhoff, Kaustubh R. Patil, Julia Camilleri, Susanne Weis

## Abstract

Machine learning analyses are widely used for predicting cognitive abilities, yet there are pitfalls that need to be considered during their implementation and interpretation of the results. Hence, the present study aimed at drawing attention to the risks of erroneous conclusions incurred by confounding variables illustrated by a case example predicting executive function performance by prosodic features. Healthy participants (n = 231) performed speech tasks and EF tests. From 264 prosodic features, we predicted EF performance using 66 variables, controlling for confounding effects of age, sex, and education. A reasonable model fit was apparently achieved for EF variables of the Trail Making Test. However, in-depth analyses revealed indications of confound leakage, leading to inflated prediction accuracies, due to a strong relationship between confounds and targets. These findings highlight the need to control confounding variables in ML pipelines and caution against potential pitfalls in ML predictions.

## Introduction

Prediction of cognitive performance is a central goal in neuroscience and related areas of research. Predicting cognitive performance is relevant for several reasons. Firstly, it enables the identification of individuals who may be at risk of cognitive decline or neurodegenerative diseases at an early stage [1] [2] [3] [4] [5] [6]. This, in turn, allows for preventative measures and early treatment. Secondly, predicting cognitive performance can help us understand the underlying mechanisms of cognitive function and identify potential biomarkers for cognitive abilities [7] [8]. Thirdly, it can aid in the development of personalised training programs based on an individual's cognitive capabilities [9].

With the rising number of variables potentially related to cognitive performance, methods for predicting cognitive functions also increase in complexity. Machine learning (ML) offers a way to study individual differences by inspecting many different possible influencing factors. ML is a field of artificial intelligence in which models are trained on data, allowing them to uncover intricate relationships and improve over time. It involves advanced statistical algorithms, which learn patterns from feature-target data with the aim to generalise to previously unseen data [10]. Such methods are of practical use for exploratory research in various fields because unknown, linear, but most importantly non-linear, relationships of a large number of variables can be inspected easily and fast. ML approaches are gaining more importance as they are able to predict the target value of an unseen individual using their features. For instance, when impaired prosodic abilities are related to a disorder, a ML model could be useful for early detection and diagnosis. However, application of ML can be problematic when applied inappropriately leading to inaccurate results and misleading conclusions.

One of the main challenges in ML relates to preventing models from displaying prediction values that are overly high in comparison to their actual predictive power [10] [11]. Barring other reasons, this is usually the case when information that should be kept strictly separate is unintentionally fed into the ML pipeline. This process is referred to as leakage [11] [12]. One form of leakage is the incorporation of information from confounding variables through the procedure of confound removal, i.e. confound-leakage [13]. Confounding variables share variance with both the dependent (target) and the independent (explanatory or predictive) variable. This means that they are associated with both variables in the analysis and can potentially have an impact on the relationship between them. It is desirable to remove the confounding information such that the model's predictions are not influenced by it. However, it is plausible that the standard confound removal procedure using linear regression might inadvertently introduce confounding information rather than removing it, causing confound leakage [14].

In the following, we demonstrate this issue using a specific example from our research, which aimed to predict cognitive performance based on prosodic variables. As executive functions are crucial cognitive capabilities in everyday human life and constitute a basic requirement for speech and communication [15] [16] [17]. we focused on predicting executive function performance in this particular application.

The term “executive functions” represents a heterogeneous set of distinguishable processes [18]. According to Ward, executive functions represent complex abilities, with which people optimise their performance in situations that require the organisation of a series of cognitive processes [19]. In spite of the lack of a universal definition of executive function performance and its subordinated domains [20], the grouping of working memory, inhibition, and cognitive flexibility [21] [22] is still the most popular [23].

Executive functions are of great relevance in relation to various pathologies, as their impairment can be observed in numerous neurological and psychiatric disorders [24] [25] [26] [27] [28]. For this reason, their investigation, both in healthy people and in different patient groups, constitutes a central component of research and diagnostics. Despite great efforts, examination and characterisation of executive functions have proven to be extremely difficult [29]. Not only is data acquisition time-consuming and costly, but the results are also dependent on subjective application factors, such as the qualification of the test conductor and the current condition of the person being tested. In addition, the measured performance depends on the individual's motivation.

What we can take advantage of in the context of testing EF is the knowledge about the relationship between executive functions and language: It is assumed that executive functions act as a cognitive control mechanism for the syntactic processing of sentences [30]. Moreover, a large variety of disorders in communication ability are associated with impaired executive functions, including dysarthria, aphasia, language pragmatic disturbances, and verbal reasoning impairments [15]. In addition to the symptoms shown on the linguistic levels of phonetics and phonology, morphology and syntax, semantics and pragmatics, the described aspects of the impaired language function also relate to the level of prosody.

Prosody can be defined as the totality of all acoustically perceptible forms of expression of speech [31]. Since prosody belongs to the realm of the phonetic structures of language and is not tied to the categories of lexeme, morpheme or phoneme, prosodic subfunctions belong to the class of suprasegmentals of language. Although several classifications of prosody have been proposed, four main domains can be distinguished: frequency related parameters, energy/amplitude related parameters, spectral parameters, and temporal parameters [32]. Fundamental frequency refers to the F0

frequency and is described as the middle pitch. Intensity of speech relates to loudness, whereas duration is defined as the quantity of speech [31].

Against the background of current literature regarding the connections between linguistic and cognitive processes, methods can be developed to draw conclusions about underlying cognitive performance with the help of speech variables. In particular, the analysis of prosodic features by speech samples provides advantages, as it offers a high external validity as well as time and cost efficiency compared to classical diagnostic procedures [33] [34] [35]. This is why procedures for objective speech analysis are gaining increasing popularity and are already in use in clinical diagnostics [36] [37].

Studies suggest that prosodic impairments may occur due to immature executive functions [38]. In addition, earlier patient studies have already shown a connection between right-hemispheric frontal brain damage and impairments of prosody [39] [40]. Recent studies also demonstrated a relation between suprasegmental disorders, regarding impaired executive functions, in foreign accent syndrome [41] [42]. Moreover, impaired working memory and impairment in prosody were observed in Parkinson's Disease [43], while reduced performance of fundamental frequency in connection with executive function damage was shown in frontotemporal dementia [44]. Furthermore, a link between prosody and divided attention, working memory and inhibition was shown in Autism Spectrum Disorder [45]. There is also clinical evidence that formant frequencies and Mel Frequency Cepstral Coefficients are associated with depressive disorders and potentially act as a biomarker [46] [47] [48] [49]. A relationship between prosodic performance, precisely disfluencies and inhibition in healthy participants was also reported by Engelhardt and colleagues [50].

In summary a link between deficient executive subfunctions and impaired prosodic skills is reported in different pathologies [38] [37] [48] [36]. These associations can be utilised to predict cognitive functions. However, these findings are primarily based on patient studies. Therefore, our initial aim was to test whether the reported correlations could predict cognitive performance in a healthy sample.

### Methods

## Participants

Participants were recruited at the Forschungszentrum Jülich and through social networks. Testing took place at the Forschungszentrum Jülich (Germany) in 2018. Each test session lasted between 150 to 180 minutes, depending on the participants' speed and the duration of the instructions. 231 healthy participants without a diagnosis of neurological or mental impairment were included in the present study (138 female, 93 male). The mean age of the sample at testing time was 35.2 years (standard deviation = 11.1, minimum = 20, maximum = 55). All participants were monolingual German. The sample varies regarding the level of education, ranging from participants who finished secondary school (n = 8), professional school/job training (n = 62), high school with a university-entrance diploma (n = 69), and university with a university degree (n = 92). All participants were paid an expense allowance of 50 EUR.

The study was approved by the ethics committee of Heinrich Heine University Düsseldorf under the registration number 2017064341. Informed consent was obtained from all participants. All experiments were performed in accordance with relevant named guidelines and regulations. Part of the data used in this study is publicly available upon request, as not all participants consent to data sharing [51].

## Design

The test sessions were divided into two parts: Firstly, the executive performance of the participants was assessed. Secondly, spontaneous speech performance was recorded in order to extract prosodic features from speech samples.

The executive function performance was assessed by the computerized test batteries *Vienna Testsystem* [52] and *Psytoolkit* [53], containing common standard tests for measuring executive function performance. In total, 66 variables from 14 different assessments of executive function performance were collected: Trail Making Test (TMT) [54], Raven's Standard Progressive Matrices [55], Wisconsin Card Sorting Test [56], Tower of London [57], and Cued Task Switching [58] are related to cognitive flexibility. Performance of N-back Non-verbal [59], Non-verbal Learning Test [60], and Corsi Block Tapping Test [61] were used in relation to working memory. Inhibition was tested by Stop Signal Task [62], Simon Task [63], and Stroop Test [64]. Divided Attention Test [65], Spatial Attention Test [65], and Mackworth Clock Test [66] were used to measure divided and spatial attention as well as vigilance. An overview of the assessed tests and the exact variables from these are shown in Table 1 (see Appendix A for the descriptions of the tests).

Spontaneous speech was tested based on a collection of three different speech samples per participant. Firstly, the participants were asked to describe the *Cookie Theft Picture* [67] within 90 seconds in as much detail as possible. Secondly, the participants were asked to talk about what they had watched on television / what kind of book they had read the day before. Thirdly, the participants were asked to describe what their favourite holiday trip would look like if money and time were no limiting factors. For the narrative tasks retelling a story and fictional storytelling, they were asked to talk for five minutes. Participants conducted all tests via a laptop, an external keyboard, and a headset-microphone.

Table 1
Assessed executive function variables adapted from Amunts et al. [68] [69]

| Test | Abbre-viation | Variables |
|---|---|---|
| COGNITIVE FLEXIBILITY | | |
| Trail Making Test | TMT | Processing time part A, Processing time part B, Difference part B-A [seconds], Quotient B/A, Errors part A, Errors part B |
| Raven's Standard Progressive Matrices | SPM | Correct items, Processing time |
| Wisconsin Card Sorting Test | WCST | Number of errors, Number of perseveration errors, Number of errors (non perseveration), Timeouts |
| Tower Of London | TOL | Planning ability, Number of correct responses, Changed his/her mind, self-correction, Choice of wrong pole, Choice of blocked pole, Choice of impossible position |
| Cued Task Switching | SWITCH | Number of errors, Timeouts, Errors of items which are incongruent |
| WORKING MEMORY | | |
| N-back Non-Verbal | NBN | Correct items, Number of commission errors, Number of errors, Mean reaction tine of correct items [seconds], Mean reaction time of errors [seconds] |
| Non-Verbal Learning Test | NVLT | Sum of correct responses, Sum of false responses, Sum of difference between correct minus false responses, Processing time |
| Corsi Block Tapping Test | CORSI | Block span, Correct items, False items, Missed items, Sequency errors |
| INHIBITION | | |
| Stop Signal Task | INHIB | Reaction time [seconds], Mean stop signal delay [seconds], Stop signal reaction time [seconds], Number of commission errors, Number of ommission errors |
| Simon Task | SIMON | Number of errors in compatible items, Number of errors in incompatible items |
| Stroop Test | STROOP | Reading interference [seconds], Naming interference [seconds], Interference-difference [seconds], Number of false reactions (reading-baseline), Number of false reactions (naming-baseline), Number of false reactions (reading-interference), Number of false reactions (naming-interference), Processing time |
| ATTENTION / VIGILANCE | | |
| Divided Attention Test | WAF-G | Number of missed items (unimodall visual), Number of false alarm (unimodal visual), Mean reaction time (unimodal visual) [ms], Number of missed items (crossmodal visual/auditive), Number of |

| Test | Abbre-viation | Variables |
|---|---|---|
| | | false alarm (crossmodal visual/auditive), Mean reaction time (crossmodal) [ms] |
| Spatial Attention Test | WAF-R | Mean reaction time (unannounced items) [ms], Number of missed items (correct announced items), Mean reaction time (correct announced items) [ms], Number of missed items (wrong announced items), Mean reaction time (wrong announced items) [ms], Mean reaction time (short SOA) [ms], Mean reaction time (long SOA) [ms], Number of errors |
| Mackworth Clock Test | MACK | Number of missed jumps, Number of false alarms |

## Feature extraction

To generate the prosodic features from the audio files collected from the speech tasks, the toolbox openSmile (**open**-**S**ource **M**edia **I**nterpretation by **L**arge feature-space **E**xtraction) [70], version 2.1.3 was used to extract the suprasegmental parameters. Although the extraction and analysis of prosodic parameters for research purposes have been done for decades in various fields and is currently a topic of big interest in the context of speech biomarkers in different pathologies [33] a lack of standardisation and thus comparability was observed [70]. The benefit of using the open-source toolbox OpenSmile is its standardised automatic computation of the prosodic features resulting in a fixed feature set. It offers the extraction of prosodic features within a set that corresponds to the main categories frequency (representing the fundamental frequency), energy/amplitude (representing the intensity), spectral parameters, and temporal parameters (representing the duration). The choice of parameters was guided by the criteria of potentially indexing physiological changes in voice production and its theoretical significance in previous literature [32]. The feature set *extended Geneva Minimalistic Acoustic Parameter Set* (eGeMAPS) was chosen, which contains 88 prosodic features. In order to keep the extraction comparable, the first 90 seconds from each audio file were chosen as input. As there are three audio samples per participant, a total of 264 prosodic features were generated per participant. All features were z-scored, i.e. the mean value was removed, and the variance was scaled to one unit. An overview of the extracted features and their descriptions, as well as the corresponding prosodic category, are shown in Table 2.

Table 2
Grouped listing of the prosodic features extracted by the toolbox OpenSmile [70].

| Prosodic feature | Variables | Description |
|---|---|---|
| FREQUENCY RELATED PARAMETERS | | |
| F0semitone<br>Mean, standard deviation, percentiles, range, rising slope, falling slope | 10 | Pitch, logarithmic F0 on a semitone frequency scale, starting at 27.5 Hz (semitone 0) |
| Jitter<br>Mean, standard deviation | 2 | Deviations in individual consecutive F0 period lengths |
| F 1–3 frequency & bandwith<br>Mean, standard deviation | 12 | Centre frequency of 1., 2., 3. formant, bandwidth of first formants 1, 2, 3 |
| ENERGY / AMPLITUDE RELATED PARAMETERS | | |
| Loudness<br>Mean, standard deviation, percentiles, range, rising slope, falling slope | 10 | Estimation of perceived signal intensity from an auditory spectrum |
| Shimmer<br>Mean standard deviation | 2 | Difference in peak amplitudes of consecutive F0 periods |
| Harmonics to Noise Ratio<br>Mean, standard deviation | 2 | Relation of energy in harmonic components to energy in noise- like components |
| SPECTRAL PARAMETERS | | |
| Spectral Flux<br>Mean, standard deviation | 3 | Difference of the spectra of two consecutive frames |
| Mel Frequency Cepstral Coefficients 1–4<br>Mean, standard deviation | 16 | Perceived pitch of the frequency spectrum |
| Harmonic differences<br>Mean, standard deviation | 4 | Ratio of energy of the first F0 harmonic (H1) to the energy of the second F0 harmonic (H2) / to the energy of the highest harmonic in the third formant range (A3) |
| Alpha Ratio<br>Mean, standard deviation | 3 | Ratio of summed energy from 50-1000 Hz and 1–5 kHz |
| Hammerberg Index<br>Mean, standard deviation | 3 | Ratio of the strongest energy peak in the 0–2 kHz region to the strongest peak in the 2–5 kHz region |

| Prosodic feature | Variables | Description |
|---|---|---|
| Spectral Slopes<br>Mean, standard deviation | 6 | Linear regression slope of the logarithmic power spectrum in the specified bands |
| F 1–3 Energy<br>Mean, standard deviation | 6 | Formant 1, 2, and 3 relative energy |
| TEMPORAL PARAMETERS | | |
| Loudness peaks per second | 1 | Number of volume highlights per second |
| Voiced segments<br>Mean, standard deviation | 3 | Amount of continuously voiced regions |
| Unvoiced segments<br>Mean, standard deviation | 2 | Amount of the continuously unvoiced regions |
| Equivalent Sound Level | 1 | Sound pressure level which has the same total energy as the actual fluctuating noise |

## Machine learning and statistical analyses

Data management and analysis were performed using Python 3 [71]. A ML approach was applied to the data following the machine learning library JuLearn [72]. The 264 extracted prosodic feature variables were specified as features and the 66 executive function variables as targets. The initial goal of our analyses was to predict each of the executive function targets using all of the prosodic features.

Firstly, cross-validation was used to determine the model performance. In cross-validation, the data set is randomly partitioned into equally sized folds. All folds except for one, are used for training the model. The hold-out fold is then used to determine the trained model's performance on unseen data. This process is repeated once for each fold as the validation fold. Then, the average of the validation performances is calculated [73]. Cross-validation was applied with ten folds (Fig. 1). Since all of the prosodic features were used to predict each of the 66 targets, 66 independent cross-validation models were performed.

In order to keep the folds balanced, stratification by target was implemented into the cross-validation pipeline, meaning that the different folds approximately followed the same distribution of the respective target [14]. Stratification can usually improve the success of model training by ensuring that the training and test data have similar distribution which reduces the risk of bias or error in the evaluation of the model. Knowing the influence of different demographic aspects on prosodic performance [74] [75] we regressed out the effects of the confounding variables sex, age, and education from the features with a linear regression model. This is standard practice since the goal is to shed light on the relationship between executive functions and prosodic features, independently of factors that are additionally related to the constructs [76] [12].

There are several regression models to choose from for usage in machine learning approaches. With his theorem *No Free Lunch* Wolpert postulated that there is no general best machine learning algorithm for all predictive modeling problems such as classification and regression [77]. We chose the Random Forest Regressor as it has already demonstrated to predict executive functions in previous studies[69] [78][79] and is commonly used[80] [81]. Random Forest is an ensemble estimator that fits a number of decision trees on various sub-samples of the dataset and uses averaging to improve the predictive accuracy and to control over-fitting. The decisions made by each tree carry equal weight, while the order of the decisions is random [82].

Following Poldrack et al. [83], accuracy was assessed by the coefficient of determination ($R^2$) [84], which measures how well the regression predictions approximate the real data points. It can be interpreted as the proportion of the variance in the dependent variable that is predictable from the independent variables. $R^2$ ranges from 0 to 1, where 1 indicates that the regression model perfectly predicts the data. In cases of negative values, the mean of the data alone fits the results better than the predicted values. Thus, negative values mean a very poor generalisation of the model. For the cross-validation results, the mean of $R^2$ was calculated over 10 folds.

Secondly, the aim of our study was to investigate which of the many prosodic features were important in connection to all features to train the model successfully. For this purpose, the feature importance was calculated by the impurity-based feature importance of Random Forest, also known as the Gini importance [85] [86]. When building a decision tree, features are selected at each node in order to divide the data into subsets that are as “pure” as possible with regard to the target variable. Gini Impurity measures how often a randomly chosen data point within a subset would be incorrectly labeled, reflecting the degree of disorder or „impurity” within the data. In contrast, Gini Importance assesses the overall decrease in node impurity resulting from splits based on a specific feature. It considers the probability of reaching each node and calculates the weighted reduction in impurity. Features with higher Gini importance are considered more important for predicting the target variable [85]. Feature importance was computed for the final estimator, as well as for each fold to estimate the variability of the importance. The sum of all feature importance scores adds up to 1.

Thirdly, detailed analyses were conducted to examine the effects of confound removal and stratification. Here, we used other models such as Random Forest Regressor, ExtraTree Regressor, and Ridge Regression to regress out the confounds from the features in order to compare model performance depending on how the confounds were removed.

Moreover, we employed an approximate permutation test approach, suggested by North and colleagues [87], to disentangle predictive information of the features from that of the confounds. To achieve this, we permuted each feature separately. Here, the association between features and targets is randomised, while the association between confounds and targets remains unchanged. 10-fold cross-validation was performed for each permutation, and $R^2$ scores for 1000 permutations were used to construct an empirical null distribution, from which p-values were computed as the proportion of permuted $R^2$ scores greater than or equal to the $R^2$ score of the original non-permuted data. The threshold value for the two-tailed test was set to $p = 0.05$. Significant p-values indicate that predictive information stems from the features rather than the confounds alone.

## Results

In cross-validation, the models were trained to predict each of the EF targets using all of the prosodic features. Regression of the confounding features sex, age, and education, and stratification by target distribution were performed. Evaluation was estimated using the coefficient of determination $R^2$ averaged over the 10 folds.

Out of 66 executive function targets, 53 variables did not show positive $R^2$ values, indicating no predictive power for these targets using our modeling approach. 13 executive function targets showed positive $R^2$ values (Fig. 2). However, only two targets, TMT BTA (processing time part A) and TMT BTB (processing time part B), showed $R^2$ values > 0.1, representing a reasonable model fit. The described TMT variables belong to the cognitive flexibility domain. An overview of $R^2$ of all 66 EF targets can be found in the supplements.

Feature importance was calculated in order to determine which of the prosodic features were particularly important for successfully predicting the EF targets. Since we observed good prediction performance ($R^2 > 0.1$) for TMT BTA and TMT BTB, we only computed feature importance for these targets. Figures 3 and 4 present the ten most important features predicting the EF targets TMT BTA and TMT BTB (see Appendix B for the feature importance of all prosodic variables). The majority of features identified as most important belong to the spectral prosodic domain. The most frequently appearing prosodic features were the Mel Frequency Cepstral Coefficients.

For the purpose of validation, we contrasted the effects of confound removal and stratification on the prediction performance for the targets TMT BTA and TMT BTB. To begin with, we compared the prediction results with the performance of the cross-validation model without regressing out the confounding variables sex, age, and education. These results indicated a worse prediction compared to the results with confound removal. Results are displayed in Fig. 5. For both TMT targets, prediction performance decreased when not removing the confounding variables. This is true for the stratified set up, as well as for the non-stratified set up. Prediction performance also decreases when not stratifying the cross-validation folds.

To explore the mechanism behind the decrease in prediction performance for the pipeline without confound removal further, and to examine whether it is related to the specific confound removal model used, we exchanged the standard confound removal model Linear Regression with other models, such as Random Forest Regressor, ExtraTree Regressor and Ridge Regression. As demonstrated in Fig. 6, the prediction performance varies depending on the choice of the confound removal model. The pipelines with the confound removal models Linear Regression and Ridge Regression indicate higher $R^2$ values than the pipelines with the confound removal models Random Forest Regressor and ExtraTree Regressor.

Finally, we evaluated the conditions with different confound removal models by using permutation tests. For the EF target TMT BTA with the cross-validation regressor Random Forest and the confound removal model Random Forest $R^2$ of 0.057 is significant ($p = 0.001$). For the EF target TMT BTB with the cross-validation regressor Random Forest and the confound removal model Ridge Regression $R^2$ of 0.196 is significant ($p = 0.032$) such as with the cross-validation regressor Random Forest and the confound removal model Linear Regression $R^2$ of 0.205 ($p = 0.017$). As shown in Table 3, all other positive prediction performances, measured by $R^2$ values, are not significant.

Table 3
Comparison of different confound removal models complemented by the p-value. CRmodel = Confound removal model, RF = Random Forest Regressor, ET = ExtraTree Regressor, LG = Linear Regression, Ridge = Ridge Regression, withoutCR = without confound removal, strat = stratified.

| **TMT BTA** | | | **TMT BTB** | | |
|---|---|---|---|---|---|
| **Condition** | **$R^2$** | **p-value** | **Condition** | **$R^2$** | **p-value** |
| CRmodelRF | -0.142 | 0.009 | CRmodelRF | -0.343 | 0.161 |
| CRmodelRF_strat | 0.057 | 0.001 | CRmodelRF_strat | -0.171 | 0.069 |
| CRmodelET | -0.172 | 0.001 | CRmodelET | -0.156 | 0.001 |
| CRmodelET_strat | -0.003 | 0.005 | CRmodelET_strat | -0.082 | 0.001 |
| CRmodelRidge | 0.097 | 0.691 | CRmodelRidge | 0.106 | 0.058 |
| CRmodelRidge_strat | 0.262 | 0.188 | CRmodelRidge_strat | 0.196 | 0.032 |
| CRmodelLG | 0.102 | 0.633 | CRmodelLG | 0.081 | 0.162 |
| CRmodelLG_strat | 0.260 | 0.200 | CRmodelLG_strat | 0.205 | 0.017 |

To summarise, we initially found a moderate predictive power of TMT BTA and TMT BTB by prosodic features. However, considering all results, there is a decrease in predictive power when not removing the confounding variables sex, age, and education, indicating confound leakage. In addition, the predictive power increases when stratification is performed. Pipelines with different models for removing confounding factors perform differently. Ultimately, two out of 20 models are significant, which suggests that the prediction is at least partly driven by the features in these models.

## Discussion

This study is based on an investigation of the relationship between executive functions and prosody through examining whether prosodic features can predict executive functions. In summary, we preliminary found a moderate predictive power of prosodic features for TMT BTA and TMT BTB. However, considering all results, there is a decrease in predictive power when not removing the confounding variables sex, age, and education, indicating confound leakage for most of the models.

Firstly, we evaluated 66 models, each predicting one executive function variable from the prosodic features. We employed 10-fold cross-validation with stratification by target variable and confound removal of sex, age, and education. The results showed poor or no prediction performance for 64 out of 66 EF targets.

Only the models for the TMT targets TMT BTA and TMT BTB, relating to cognitive flexibility, initially appeared to have a moderately valid predictive performance. Without the additional analyses that we conducted for validation, these results could be interpreted as follows: Our results would have confirmed findings from previous studies on a narrow correlation between executive functions and language in general [88] [17], and would have been in line with research conducted in different patient cohorts [43] [45] [50], reporting connections between cognitive flexibility and prosody [34]. In our study, we would have found these associations in healthy participants. Consistent with the literature, this study would have shown that features from various prosodic domains are important for the models to learn. This would have validated that prosodic features of different kinds are closely related to executive functions, as described in previous studies [89] [90] [91]. Furthermore, predominantly spectral prosodic parameters would have shown importance for the model fits, especially the Mel Frequency Cepstral Coefficients, which are already used as a biomarker in depressive disorders [47] [49]. As described in Table 2, the Mel Frequency Cepstral Coefficients are defined as the perceived pitch of the frequency spectrum. More precisely, these are coefficients of the Mel scale, which relates the perceived frequency of a tone to the actual measured frequency. It scales the frequency in order to match more closely what the human ear can hear [92]. It therefore would have been deduced from the study that spectral parameters, in particular the Mel Frequency Cepstral Coefficients, are closely related to executive functions. Furthermore, the findings would have confirmed that easy-to-capture spontaneous speech derived from different tasks is suitable for the extraction of prosodic features. In summary, the present research would have raised the possibility that this predictive power of prosodic features could be an important biomarker for executive function impairment or its future decline.

However, given the additional in-depth analyses of the ML pipeline that partly invalidate the initial results, our findings need to be reinterpreted as follows:

We expect models to perform better if the effects of the confounding variables are not excluded, given that this would provide more information for the algorithm to learn. However, the prediction performance decreases for both TMT targets when not removing the confounding variables sex, age, and education. This is not in line with our expectation because in our scenario, the prediction performance should be worse if the confounding variables are removed, as the algorithm can then only learn from the association between confound-free features and the target. We found that information from these confounds, namely sex, age, and education leaked into the predictions through the confound removal procedure. The inadvertent injection of this information occurs particularly when the confounding variables and the targets show a strong correlation and this is coupled with the use of a high number of features, as explained by Hamdan et al. [13] and Sasse & Nicolaisen-Sobesky et al. [12]. This is indeed the case in our data set (see Appendix C). There is a strong correlation between the TMT targets and the confounding variables. In addition, we use a high number of features within the cross-validation pipeline, because we wanted to investigate EF and prosody in an exploratory manner. While our data set was relatively small compared to most ML studies, which typically increases the risk of leakage [93], it represents a reasonable size when compared to studies investigating speech biomarkers [33]. The results also confirm that these observations occur in both stratified and non-stratified conditions. As expected, it can be shown that stratification by target distribution generally increases the predictive performance. This is in line with Diamantidis et al. [94] and Hastie et al. [14], who show that equally representative cross-validation folds lead to improved predictive power. Additionally, it is demonstrated that stratification can also increase confound leakage This can be derived from the fact that the difference in predictive power between the pipelines with and without confound removal is even greater in the stratified condition (Fig. 6). Furthermore, the results illustrate that the observed confound leakage is not bound to the use of Linear Regression as the confound removal model, but also occurs when other models are employed.

Overall, these observations raise concerns about the trustworthiness of the primary results. Nonetheless one cannot definitively rule out whether information from the features also influenced the predictive power of the present results. We, therefore, conducted permutation testing for the different cross-validation models. Since the permutation tests for the two TMT targets each identified models that can be interpreted as significant, we speculate that predictive power is partly due to the information contained in the features despite the confounding variables also contributing to the prediction. However, this was only observed in two of 66 EF targets and for these two targets only in specific confound removal models. For this reason, we only conditionally derive the predictive power of prosodic features. Further analyses of this type with other data sets would need to be carried out to verify this.

In conclusion, the present results highlight the challenges and pitfalls when conducting ML analyses with the aim of predicting variables of interest including cognitive performance. This example shows which misinterpretations could have been deduced from the initial results. This can be particularly dangerous if the findings match previous studies, as in the case here. This is crucial, as ML studies are becoming increasingly important and widely employed, especially with the accessibility of large amounts of data. In this respect, we caution and recommend that when using ML analyses to predict cognitive performance, quality controls should be performed to prevent false results. This is also true when interpreting ML results of other researchers. This study has contributed to uncovering more insight into a pitfall in ML analysis arising due to confound leakage. As confounding is ubiquitous in social and biological sciences, it should be further deciphered how confound leakage occurs and which contributing factors can be taken into account. Additionally, our analysis framework provides a blueprint for further research investigating whether prosody can serve as a predictive biomarker of executive dysfunction.

## Declarations

#### Data Availability

Part of the data used in this study is publicly available upon request. Researchers who wish to acquire access to the data are kindly asked to contact Julia A. Camilleri at spexdata@fz-juelich.de, as described in the related publication Camilleri, J.A., Volkening, J. et al. SpEx: a German-language dataset of speech and executive function performance. Sci Rep 14, 9431 (2024). https://doi.org/10.1038/s41598-024-58617-3

#### Acknowledgements

This study was supported by

- the Deutsche Forschungsgemeinschaft (DFG, GE 2835/2–1, EI 816/16-1 and EI 816/21-1),
- the National Institute of Mental Health (R01-MH074457),
- the Helmholtz Portfolio Theme "Supercomputing and Modeling for the Human Brain",
- the Virtual Brain Cloud (EU H2020, no. 826421) &
- the National Institute on Aging (R01AG067103).

#### Author information

These authors contributed equally: Julia A. Camilleri and Susanne Weis.

**Institute of Systems Neuroscience, Medical Faculty, Heinrich Heine University Düsseldorf, Düsseldorf, Germany.**

Gianna Kuhles, Sami Hamdan, Simon B. Eickhoff, Kaustubh R. Patil, Julia A. Camilleri, Susanne Weis

**Institute of Neuroscience and Medicine, Brain and Behaviour (INM-7), Research Centre Jülich, Jülich, Germany.**

Gianna Kuhles, Sami Hamdan, Simon B. Eickhoff, Kaustubh R. Patil, Julia A. Camilleri, Susanne Weis

**Department of Psychiatry, Psychotherapy and Psychosomatics, Medical Faculty, RWTH Aachen University, Aachen, Germany.**

Stefan Heim

**Institute of Neuroscience and Medicine, Structural and functional Organization of the Brain (INM-1), Research Center Jülich, Jülich, Germany.**

Stefan Heim

#### Author contributions

G.K., J.A.C., S.W. conceived the project and designed the study. S.H., S.H., S.B.E., K.R.P. contributed essential resources. G.K. with contributions from S.W. and all other authors wrote the manuscript.

#### Competing interests

The authors declare no competing interests.

## Figures

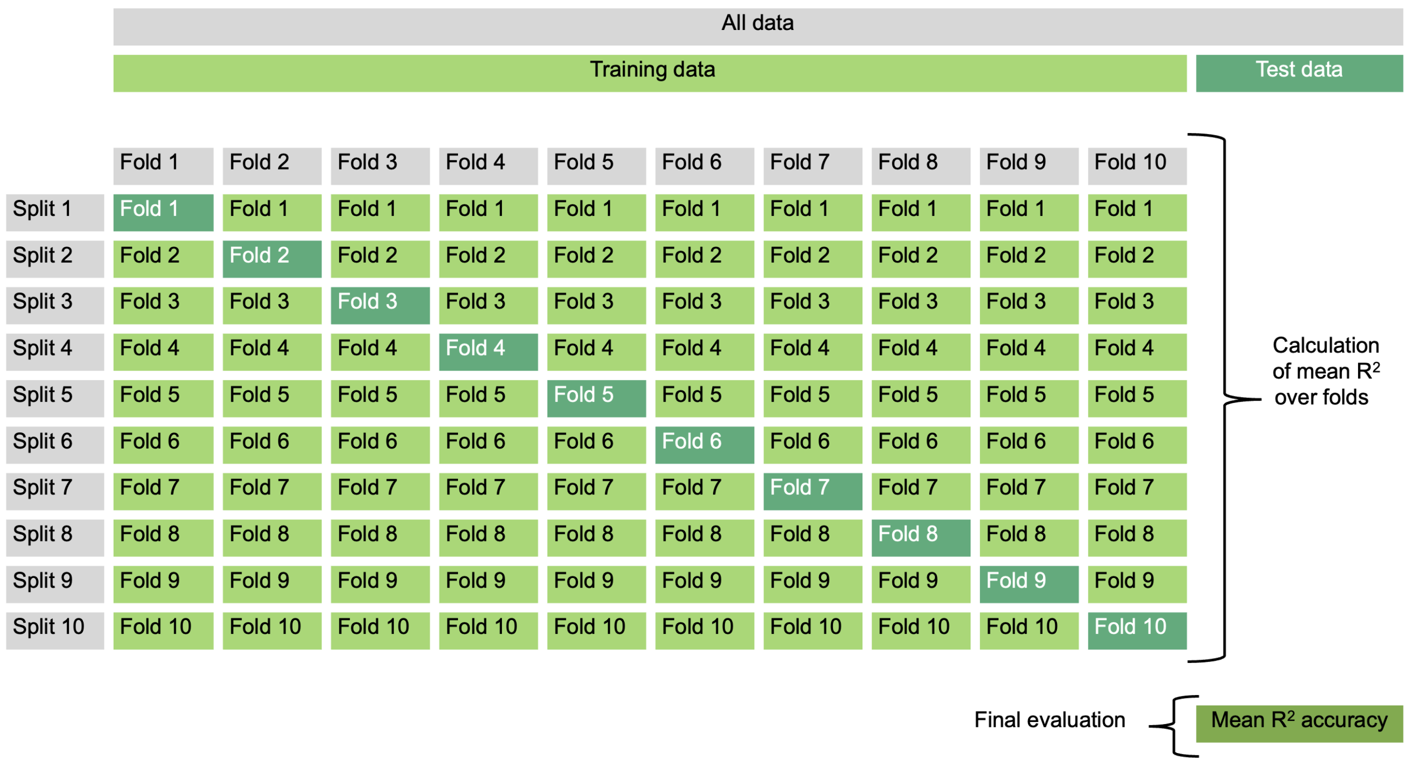

### Figure 1

10-fold cross-validation design for each executive function target.

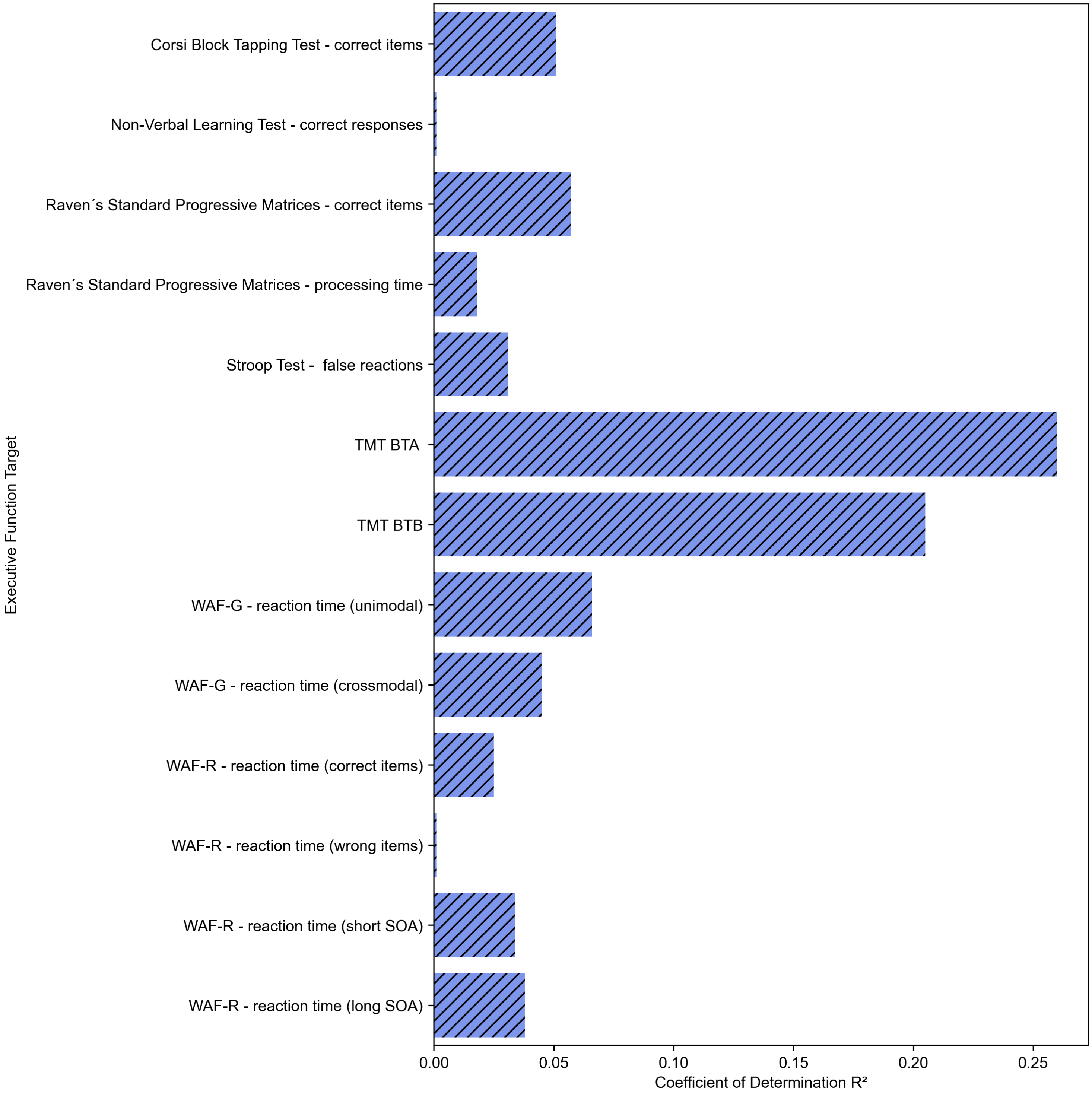

### Figure 2

Prediction of executive function targets by prosodic features.

Cross-validation model with confound removal and stratified by target distribution. Only targets with positive $R^2$ values are displayed. TMT BTA = Trail Making Test - processing time part A, TMT BTB = Trail Making Test - processing time part B.

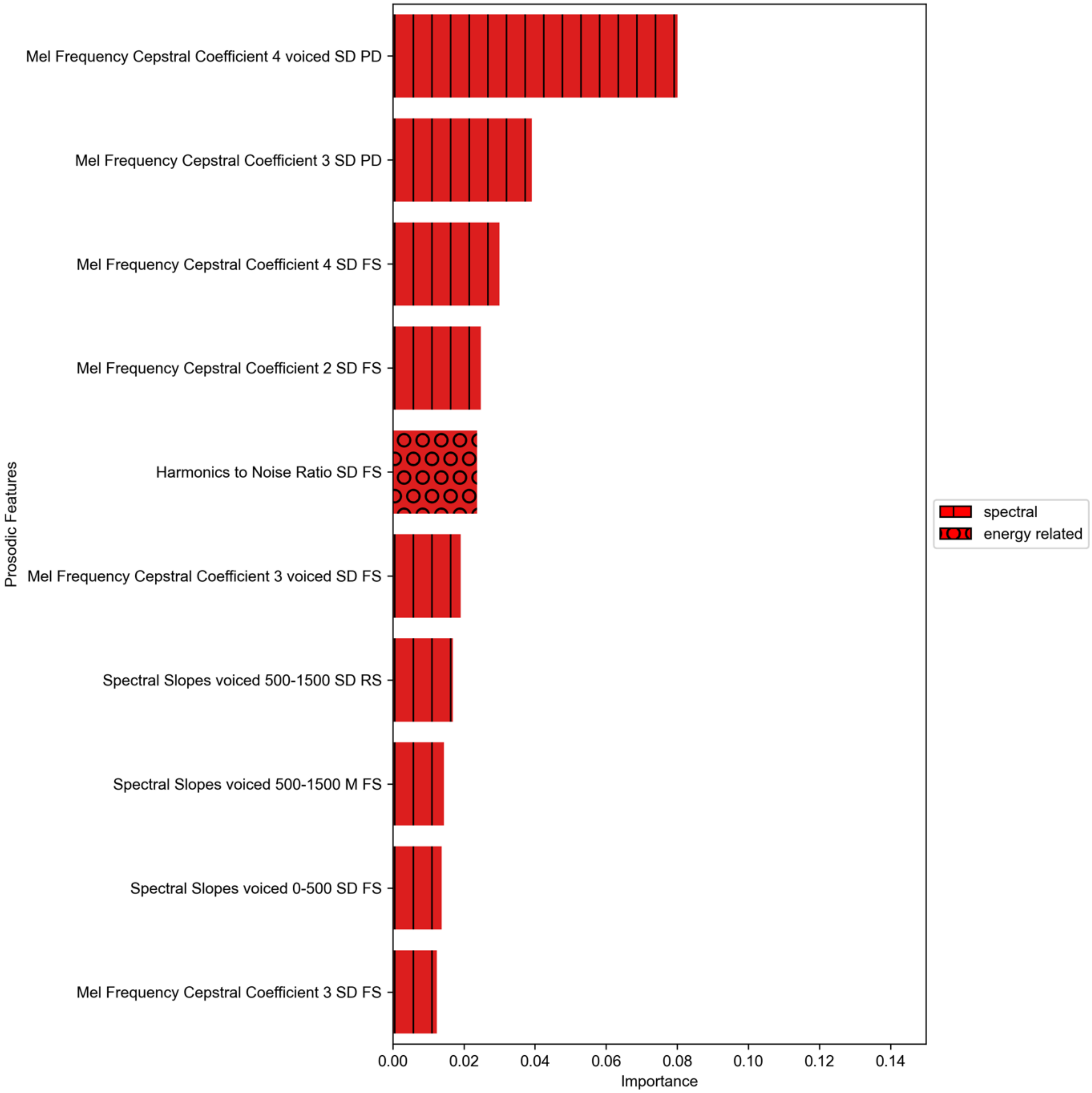

### Figure 3

Feature importance for TMT BTA.

TMT BTA = Trail Making Test - processing time part A, SD = standard deviation, M = mean, PD = picture description, RS = retelling a story, FS = fictional storytelling.

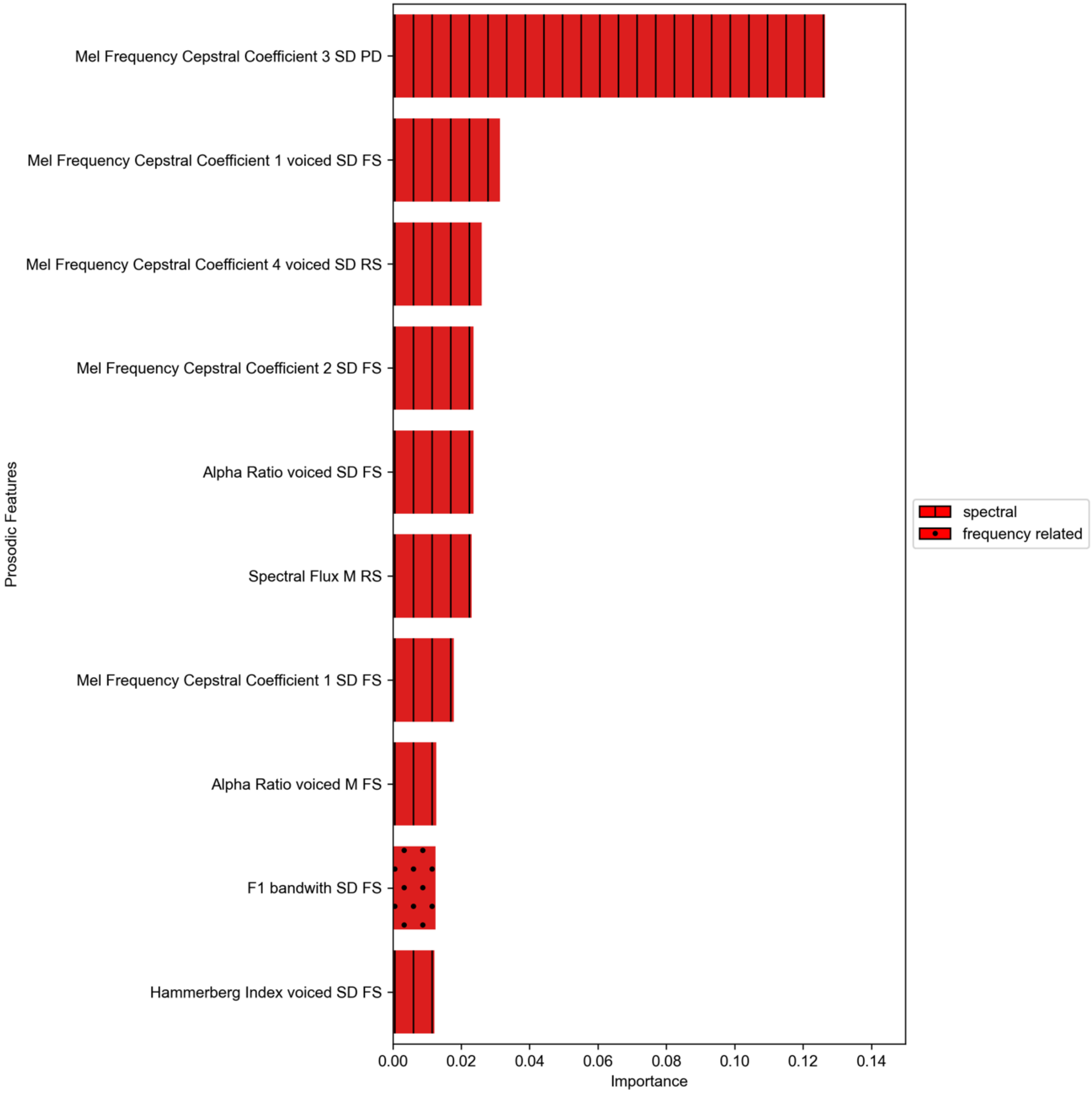

### Figure 4

Feature importance for TMT BTB.

TMT BTB = Trail Making Test - processing time part B, SD = standard deviation, M = mean, PD = picture description, RS = retelling a story, FS = fictional storytelling.

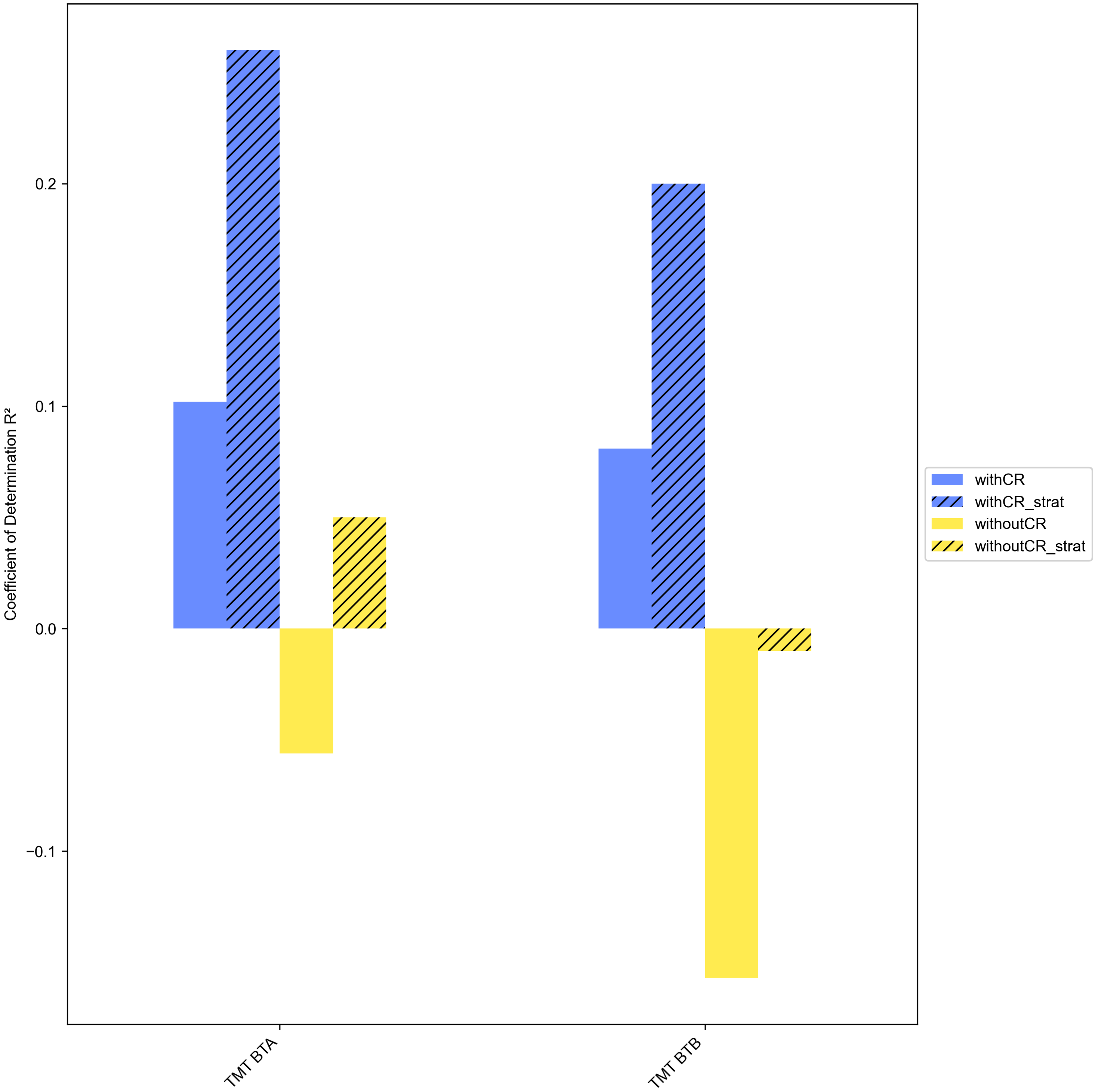

### Figure 5

Prediction of TMT targets in different conditions regarding confound removal and stratification. TMT BTA = Trail Making Test - processing time part A, TMT BTB = Trail Making Test - processing time part B, confounding variables (sex, age, education) and stratification: with CR = with confound removal, strat = stratified, without CR = without confound removal.

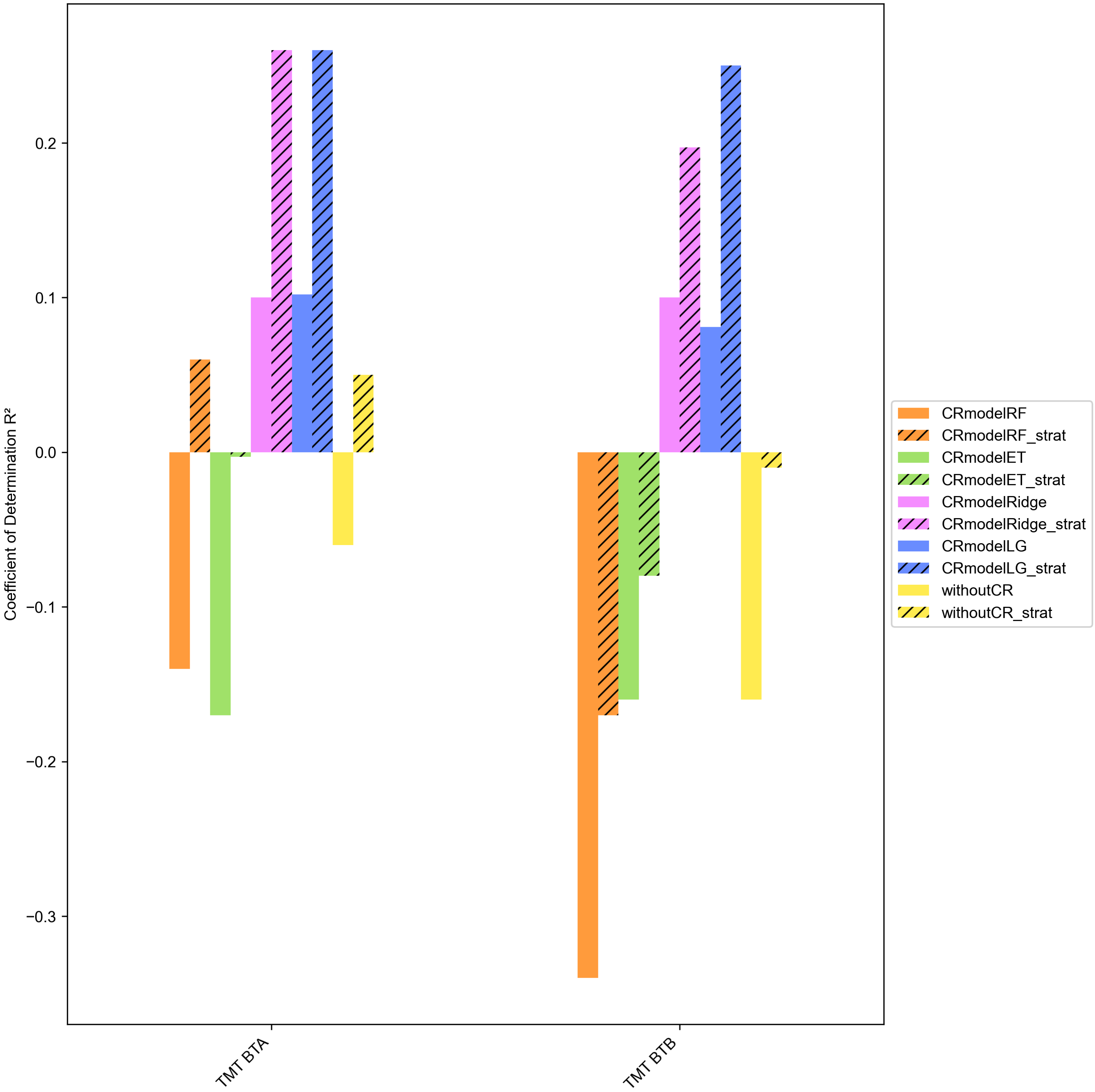

### Figure 6

Prediction of TMT targets in different conditions regarding different confound removal models. TMT BTA = Trail Making Test - processing time part A, TMT BTB = Trail Making Test - processing time part B, confounding variables (sex, age, education) and stratification. CRmodel = Confound removal model, RF = Random Forest Regressor, ET = ExtraTree Regressor, Ridge = Ridge Regression, LG = Linear Regression, withoutCR = without confound removal, strat = stratified.

## Supplementary Files

This is a list of supplementary files associated with this preprint. Click to download.

- AppendixPitfallsinusingMLtopredictcognitivefunctionperformance.docx
